# Quantitative Multi-Parameter Mapping Optimized for the Clinical Routine

**DOI:** 10.3389/fnins.2020.611194

**Published:** 2020-12-07

**Authors:** Graham Cooper, Sebastian Hirsch, Michael Scheel, Alexander U. Brandt, Friedemann Paul, Carsten Finke, Philipp Boehm-Sturm, Stefan Hetzer

**Affiliations:** ^1^Experimental and Clinical Research Center, Max Delbrueck Center for Molecular Medicine and Charité–Universitätsmedizin Berlin, Corporate Member of Freie Universität Berlin, Humboldt-Universität zu Berlin, Berlin Institute of Health, Berlin, Germany; ^2^NeuroCure Clinical Research Center, Charité–Universitätsmedizin Berlin, Corporate Member of Freie Universität Berlin, Humboldt-Universität zu Berlin, Berlin Institute of Health Berlin, Berlin, Germany; ^3^Einstein Center for Neurosciences Berlin, Charité - Universitätsmedizin Berlin, Corporate Member of Freie Universität Berlin, Humboldt-Universität zu Berlin, and Berlin Institute of Health, Berlin, Germany; ^4^Department of Experimental Neurology and Center for Stroke Research Berlin, Charité - Universitätsmedizin Berlin, Corporate Member of Freie Universität Berlin, Humboldt-Universität zu Berlin, and Berlin Institute of Health, Berlin, Germany; ^5^Berlin Center for Advanced Neuroimaging, Charité–Universitätsmedizin Berlin, Corporate Member of Freie Universität Berlin, Humboldt-Universität zu Berlin, Berlin Institute of Health Berlin, Berlin, Germany; ^6^Bernstein Center for Computational Neuroscience, Humboldt-Universität zu Berlin, Berlin, Germany; ^7^Department of Neuroradiology, Charité–Universitätsmedizin Berlin, Corporate Member of Freie Universität Berlin, Humboldt-Universität zu Berlin, Berlin Institute of Health Berlin, Berlin, Germany; ^8^Department of Neurology, University of California, Irvine, Irvine, CA, United States; ^9^Department of Neurology, Charité–Universitätsmedizin Berlin, Corporate Member of Freie Universität Berlin, Humboldt-Universität zu Berlin, Berlin Institute of Health Berlin, Berlin, Germany; ^10^Berlin School of Mind and Brain, Humboldt-Universität zu Berlin, Berlin, Germany; ^11^NeuroCure Cluster of Excellence and Charité Core Facility 7T Experimental MRIs, Charité Universitätsmedizin Berlin, Corporate Member of Freie Universität Berlin, Humboldt-Universität zu Berlin, Berlin Institute of Health Berlin, Berlin, Germany

**Keywords:** quantitative multi-parameter mapping, intra-subject reliability, Gibb's ringing, quantitative MRI, signal-to-noise-ratio

## Abstract

Using quantitative multi-parameter mapping (MPM), studies can investigate clinically relevant microstructural changes with high reliability over time and across subjects and sites. However, long acquisition times (20 min for the standard 1-mm isotropic protocol) limit its translational potential. This study aimed to evaluate the sensitivity gain of a fast 1.6-mm isotropic MPM protocol including post-processing optimized for longitudinal clinical studies. 6 healthy volunteers (35±7 years old; 3 female) were scanned at 3T to acquire the following whole-brain MPM maps with 1.6 mm isotropic resolution: proton density (PD), magnetization transfer saturation (MT), longitudinal relaxation rate (R1), and transverse relaxation rate (R2^*^). MPM maps were generated using two RF transmit field (B1+) correction methods: (1) using an acquired B1+ map and (2) using a data-driven approach. Maps were generated with and without Gibb's ringing correction. The intra-/inter-subject coefficient of variation (CoV) of all maps in the gray and white matter, as well as in all anatomical regions of a fine-grained brain atlas, were compared between the different post-processing methods using Student's *t*-test. The intra-subject stability of the 1.6-mm MPM protocol is 2–3 times higher than for the standard 1-mm sequence and can be achieved in less than half the scan duration. Intra-subject variability for all four maps in white matter ranged from 1.2–5.3% and in gray matter from 1.8 to 9.2%. Bias-field correction using an acquired B1+ map significantly improved intra-subject variability of PD and R1 in the gray (42%) and white matter (54%) and correcting the raw images for the effect of Gibb's ringing further improved intra-subject variability in all maps in the gray (11%) and white matter (10%). Combining Gibb's ringing correction and bias field correction using acquired B1+ maps provides excellent stability of the 7-min MPM sequence with 1.6 mm resolution suitable for the clinical routine.

## Introduction

Quantitative magnetic resonance imaging (qMRI) has the potential to revolutionize neuroradiology by deriving absolute measures in physical units that are independent of technical confounders and provide insight into physiologically meaningful properties of tissue (Tofts, 2010). Only quantitative measures allow a statistically valid comparison and interpretation of effect sizes across brain areas, subjects, species and sites (Sullivan and Feinn, 2012; Weiskopf et al., 2015; Chen et al., 2017). Quantitative multi-parameter mapping (MPM) is an excellent example, whereby four quantitative maps [proton density (PD), magnetization transfer saturation (MT), longitudinal relaxation rate (R1 = 1/T1), and transverse relaxation rate (R2^*^ = 1/T2^*^)] of the whole brain can be measured in a scan time of around 20 min (Weiskopf et al., 2013). These maps are sensitive to microstructural tissue properties of high clinical relevance, such as myelin and iron content (Weiskopf et al., 2015), and have been shown to be sensitive to pathology, for example in multiple sclerosis (Jurcoane et al., 2013; Gracien et al., 2017; Lommers et al., 2019) and spinal cord injury (Grabher et al., 2015). Despite the high validity of MPM (Weiskopf et al., 2013; Leutritz et al., 2020), the widely used standard MPM protocol with 1 mm resolution (Weiskopf et al., 2013; Callaghan et al., 2014; Grabher et al., 2015; Ziegler et al., 2018; Lommers et al., 2019; Leutritz et al., 2020; Taubert et al., 2020) has a relatively long acquisition time, limiting its translational potential (Bagnato et al., 2020). Particularly in the clinical context, scan-time is minimized to increase patient comfort, reduce motion artifacts (Havsteen et al., 2017), and increase cost-efficiency for a high patient throughput.

To optimize the clinical utility of MPM, we propose a modified scan protocol that seeks to find a compromise between shortened scan time and highest achievable signal-to-noise ratio (SNR). Since SNR is directly proportional to voxel volume (Edelstein et al., 1986), spatial resolution should be reduced to the lowest value that still provides discernibility for fine-grained structures of interest such as multiple sclerosis lesions (Filippi et al., 2019), while the scan-rescan variability is reduced to a level close to physiological noise where detection power for small longitudinal signal changes is optimal (Triantafyllou et al., 2011). The well-known, simplified relationship between voxel volume, V, acquisition time, T, and SNR~V·√T, indicates that even a small decrease in spatial resolution (and thus an increase in voxel volume) allows a tremendous decrease in scan time to maintain a constant SNR. For example, doubling the voxel size from 1 to 2 mm, corresponding to an increase in voxel volume by a factor of 8, results in a reduction of scan time T by a factor of 8^2^ = 64 to obtain the same SNR. Additional optimization of scan-rescan stability by correcting for artifacts like Gibb's ringing, which has a high impact on image reliability especially in the vicinity of sharp tissue boundaries (Kellner et al., 2016), has not been investigated in MPM.

Here, we present an MPM protocol based entirely on standard manufacturer sequences that can be readily translated into the clinical routine. Given the envisioned clinical application, we used a 1.6 mm isotropic MPM protocol with a clinically feasible overall acquisition time under 10 min. We investigated intra- and inter-subject variability for tissue classes and anatomical subregions using an atlas-based analysis and compared the effect of using different bias field correction methods as well as correcting for Gibb's ringing to determine the optimum post-processing strategy for this fast MPM protocol.

## Materials and Methods

### Subjects

For intra-/inter-subject variability evaluation, a sample of six healthy volunteers (3 females, age 34.7 ± 7.3 years) were scanned at the same scanner three times. Multiple scans were acquired sequentially in one session, i.e., the volunteer exited and re-entered the scanner, to simulate a naturalistic head placement as would occur in a longitudinal study. This study was approved by the local ethics committee and conducted in accordance with the Declaration of Helsinki and German law. All participants provided written informed consent.

### MRI Acquisition

All scans were acquired on a 3T MR scanner (Magnetom Prisma, Siemens Healthineers, Erlangen, Germany) using a 20-channel receive radio-frequency (RF) head-neck coil covering both the cervical spinal cord and the brain. Trained radiographers placed participants in the same position to obtain high reproducibility across participants and time points, minimizing bias related to non-linearity of the gradient and RF bias-fields over time as well as nerve fiber orientation effects relative to the static magnetic field (Sati et al., 2012). All volumes were automatically aligned to the head-base axis (van der Kouwe et al., 2005) with a 25° rotation of the sagittal plane to avoid eye-related motion artifacts or folding of the nose into the cortex (Callaghan et al., 2019).

Each 7-min long MPM scan with 1.6 mm isotropic resolution was comprised of three different 3D multi-echo fast low-angle shot (FLASH) gradient-echo acquisitions designed to provide measures of proton density (PD), magnetization transfer saturation (MT), longitudinal relaxation rate (R1 = 1/T1), and transverse relaxation rate (R2^*^ = 1/T2^*^) with a field-of-view (FOV) of 224 × 256 mm^2^ (matrix-size 140 × 160) and 112 partitions. To minimize acquisition time parallel imaging was used in the phase-encoding direction (anterior-posterior) employing a generalized auto-calibration partially parallel acquisition algorithm (GRAPPA) factor of 2 combined with a 6/8 partial Fourier acquisition in the partition direction (left-right). The readout bandwidth was 470 Hz/pixel allowing six echoes between 2.46 and 14.78 ms for all three acquisitions. Contrast parameters were: repetition time TR = 37 ms, flip angle FA = 6° for MT-weighted images with a Gaussian off-resonance RF pulse (500°, 10 ms, 1,200 Hz off-resonance, 192 Hz bandwidth) prior to non-selective excitation; TR = 18 ms, FA = 4° and 25° for PD-weighted images and T1-weighted images, respectively. In order to enable bias field correction a RF transmit (B1+) map was acquired for all runs in a FOV exactly matching the MPM scans with an isotropic resolution of 4 mm. The B1+ map was derived from spin-echo/stimulated echo acquisitions with a total scan time of 2 min per run using a standard vendor sequence (Leutritz et al., 2020).

In order to demonstrate the impact of different image resolutions on the variability of MPM data one volunteer (male, aged 42) was additionally scanned twice with isotropic resolutions of {2.67; 2.00; 1.60; 1.33; 1.00} mm, a matrix-size of {84 × 96; 112 × 128; 140 × 160; 168 × 192; 224 × 256} and {64; 88; 112; 128; 176} partitions, resulting in scan times of {2.7, 4.6; 7; 9.4; 16.5} min per run. Including the RF transmit maps acquired for each run the overall scan time was 2 h for this multi-resolution scan-rescan session.

### Quantitative Map Generation

MR parameter maps of PD, MT, R1, and R2^*^ were generated using the hMRI toolbox (Tabelow et al., 2019) version 0.2.0 (RRID:SCR_017682) implemented in SPM12 version 7219 (RRID:SCR_007037) using MATLAB 2019a (RRID:SCR_001622). Maps of MT, R1, and R2^*^ were calculated using the ESTATICS model (Weiskopf et al., 2014). Receive field inhomogeneities were corrected using Unified Segmentation (Tabelow et al., 2019). For the correction of transmit RF field (B1+) imperfections we compared the acquired B1+ map with a data driven estimation of the B1+ bias-field using the UNICORT approach by Weiskopf et al. (2011). The influence of Gibb's ringing correction as described by Kellner et al. (2016) was tested by removing the typical oscillatory patterns from all 6 echoes of the raw images (MTw, T1w, PDw) before parameter quantification with the hMRI toolbox.

### Post-processing

All four maps (MT, PD, R1, and R2^*^) were transformed to MNI space using SPM12 standard tools. The extended tissue probability map from the hMRI toolbox (Tabelow et al., 2019) with a threshold of 0.6 was used to calculate the average values and corresponding intra-/inter-subject coefficients of variation (CoV) of all four maps in the gray and white matter. Average values and the CoV of all maps in each region of the Neuromorphometrics atlas (Bakker et al., 2015) were also calculated using SPM12.

### Statistical Analysis

Statistical analyses were calculated in the gray and white matter masks and in all 60 ROIs of the Neuromorphometrics atlas. For each subject the intra-subject CoV was calculated by dividing the voxel-wise standard deviation by the mean across all three scans. The intra-subject CoV was used to analyze the impact of Gibb's ringing correction and compare two B1+ inhomogeneity correction techniques: employing acquired B1+ maps and using a data-driven approach (Weiskopf et al., 2011). Group comparisons were made using Student's *t*-test. Statistical analyses were conducted in R version 3.6.1 (www.R-project.org) and MATLAB 2019a.

## Results

### Multi-Resolution Analysis

**Figure 1:**
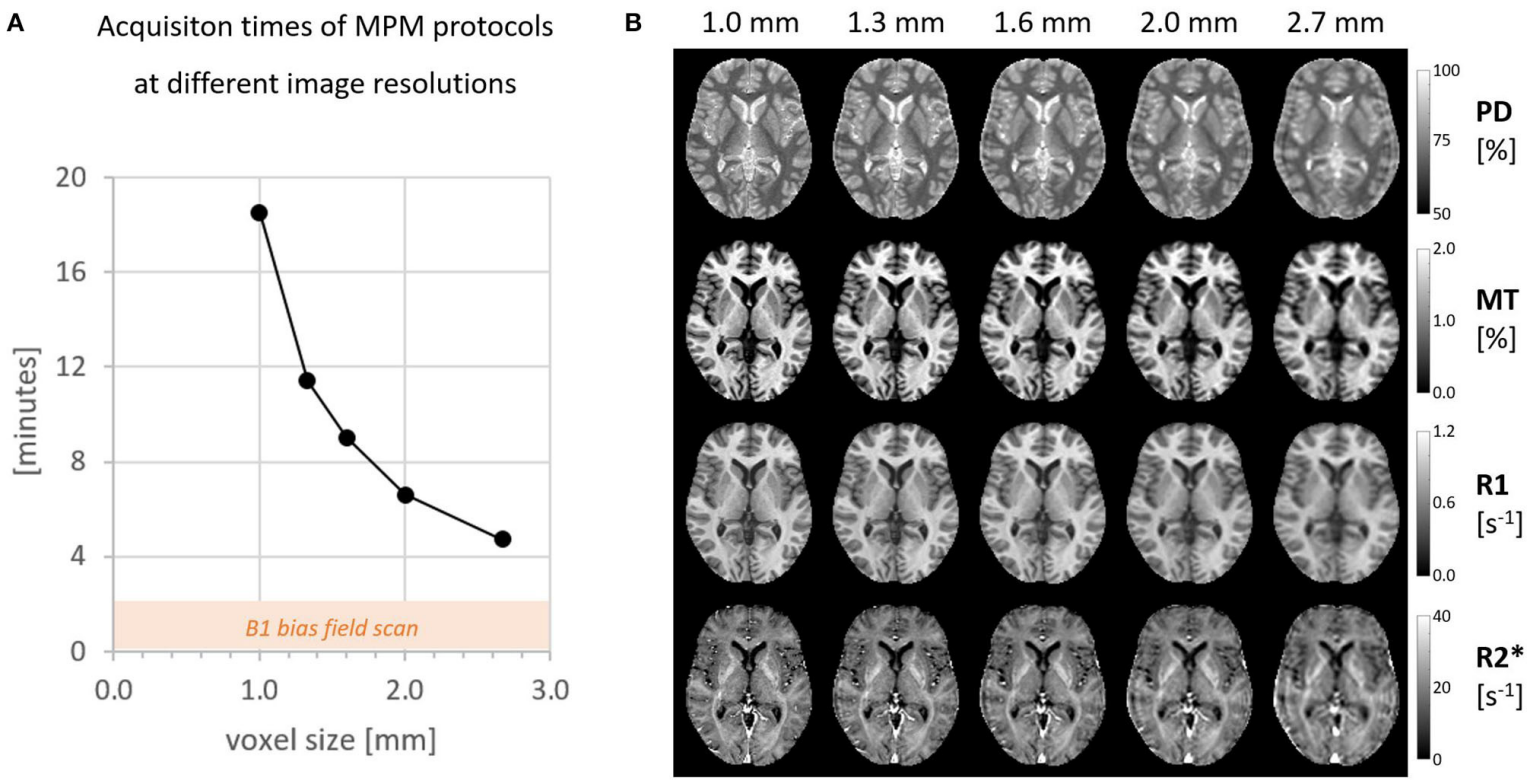
**(A)** The total scan time for one MPM session including B1+ map. **(B)** All four quantitative maps acquired at different isotropic resolutions.

Figure 1 gives an overview of the data acquisition times for all 5 image resolutions between 1 and 2.7 mm and the corresponding quantitative maps. An experienced neuroradiologist (M.S., 10 years of experience) rated the image quality of isotropic resolutions higher than 2 mm good enough to resolve common pathologies in clinical practice.

**Figure 2:**
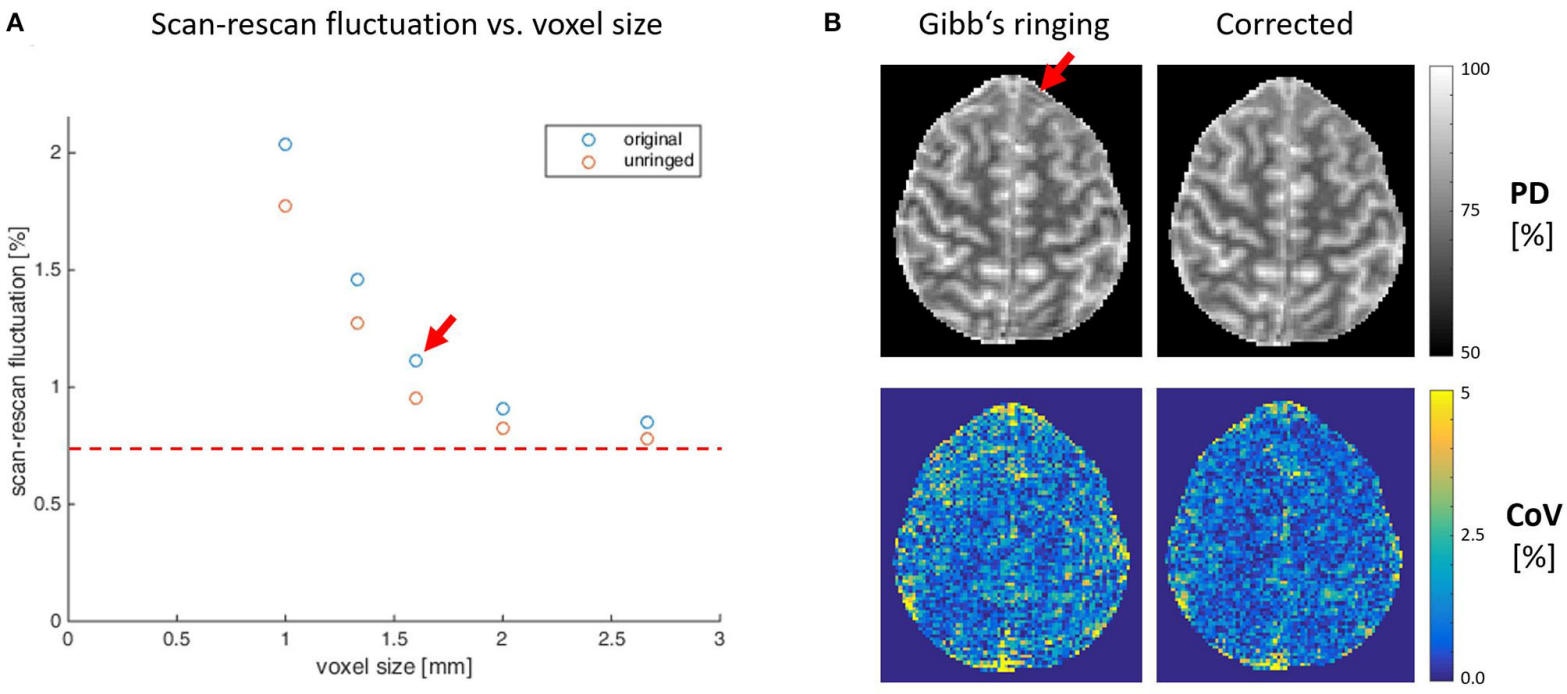
**(A)** Scan-rescan variability within the white matter at different isotropic resolutions on the example of proton density contrast. Increasing the voxel size decreases scan-rescan fluctuations until a point (red dashed line) where we assume physiological noise starts to dominate. A decrease in voxel size below 1.6 mm (red arrow) comes at the cost of longitudinal stability and longer acquisition times (compare Figure 1A), which are incompatible with the needs of clinical routine. Correcting for Gibb's ringing increases stability (blue vs. red circles), especially at higher image resolution. **(B)** One exemplary slice (1.6 mm resolution) illustrating the advantages of Gibb's ringing correction on image quality and stability, especially in areas close to sharp tissue boundaries (red arrow).

Figure 2 depicts the reduction of the CoV with increasing voxel size in the example of PD and the improvement of data quality after Gibb's ringing correction (see https://clinicalmpm.github.io for the remaining contrasts). The image resolution of 1.6 mm chosen for the multi-subject analysis is close to the optimum where physiological noise dominates (Triantafyllou et al., 2011).

### Multi-Subject Analysis

**Figure 3:**
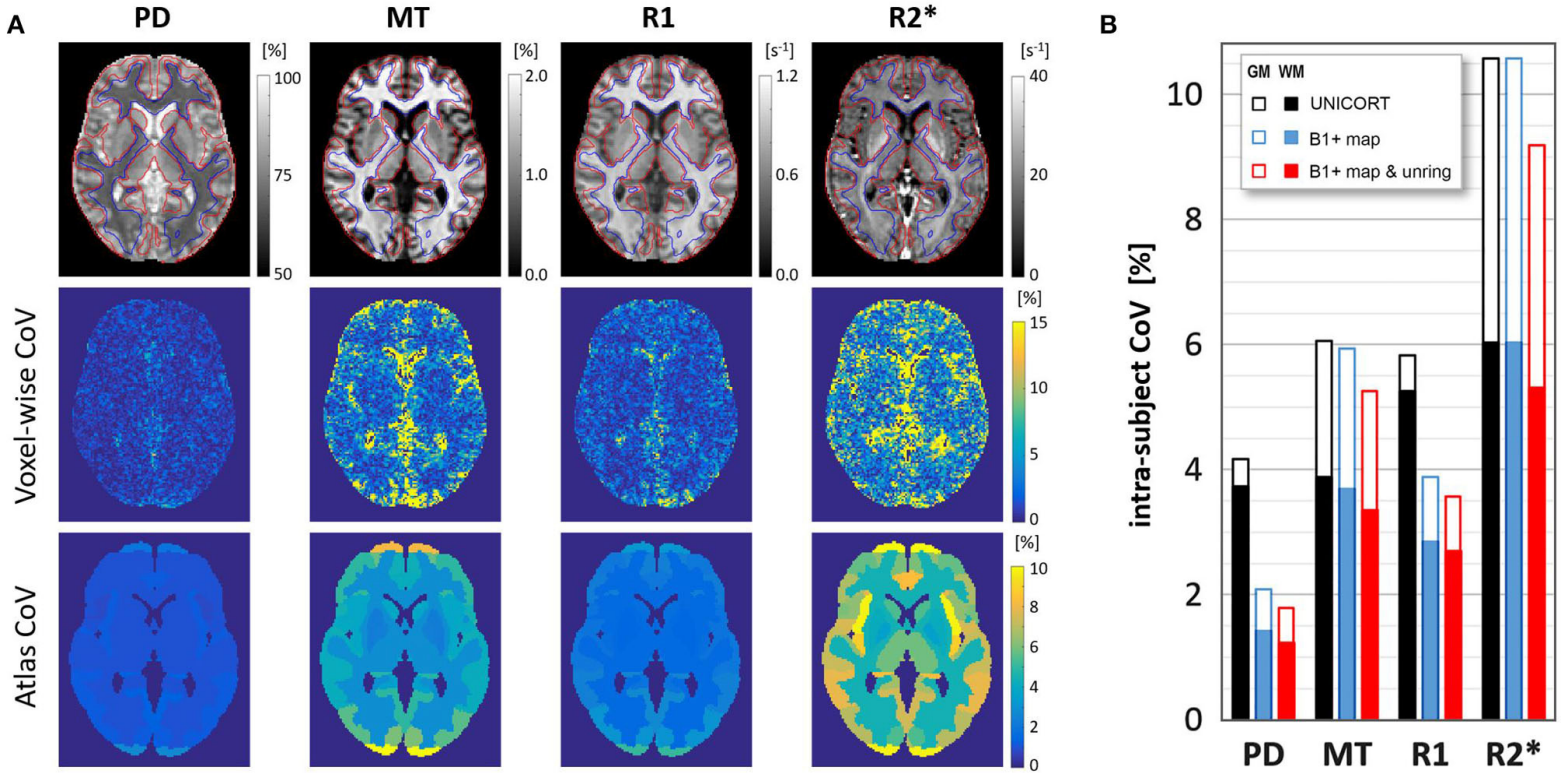
**(A)** An axial slice for a typical volunteer showing PD, MT, R1, R2* parameter maps for the optimized 1.6-mm protocol (upper row) including contour lines of white matter (blue) and gray matter (red) masks used in the analysis; intra-subject CoV across the three scans (middle row); and the average CoV of MT, PD, R1, R2* in the atlas regions - see also Table 2 and https://clinicalmpm.github.io. **(B)** The effect of B1+ map correction (data-driven UNICORT vs acquired B1+ map) and Gibb's ringing correction on the group-averaged scan-rescan variability in the gray and white matter for all 4 MPM contrasts. Note the negligible effect of using the 2-minute long B1 bias-field scan for the MT and R2* map.

High quality MPM parameter maps with absolute values comparable to those previously reported in healthy participants (Weiskopf et al., 2013; Callaghan et al., 2014; Lommers et al., 2019) were generated using the multi-subject protocol acquired with 1.6 mm resolution (see Figure 3A and Table 1). Using the data-driven B1+ correction (UNICORT) resulted in small deviations from the absolute values reported in Table 1 for gray/white matter (MT: −0.27/−0.81%, R1: 6.79/6.69%). R2^*^ and PD were not compared because R2^*^ quantification is not affected by B1+ correction in the hMRI toolbox and PD is not quantified when using UNICORT (Tabelow et al., 2019). Without Gibb's ringing correction the absolute values in the gray/white matter deviated slightly compared to Table 1. (PD: 0.46/0.10%, MT: 0.85/0.54%, R1: 0.39/0.47%, R2^*^: −0.08/0.15%).The mean parameter values and CoV in the gray and white matter are shown in Table 1 and Figure 3B, respectively. Compared to gray matter, the intra-subject CoV of each contrast showed higher stability in white matter by a factor of 1.50 ± 0.09 for PD, 1.57 ± 0.12 for MT, 1.35 ± 0.14 for R1, and 1.73 ± 0.09 for R2^*^.

**Table 1:** Group-average values and intra-/inter-subject coefficients of variation of PD, MT, R1, and R2^*^ maps using B1+ map and Gibb's ringing correction.

	**Parameter value**		**Intra-subject CoV [%]**		**Inter-subject CoV [%]**	
	**Gray matter**	**White matter**	**Gray matter**	**White matter**	**Gray matter**	**White matter**
PD [%]	78.92 ± 0.38	70.52 ± 0.40	1.85 ± 0.40	1.22 ± 0.20	0.48 ± 0.04	0.56 ± 0.05
MT [%]	0.93 ± 0.04	1.59 ± 0.04	5.26 ± 1.37	3.35 ± 0.83	4.67 ± 0.26	2.45 ± 0.15
R1 [s^−1^]	0.62 ± 0.02	0.90 ± 0.02	3.58 ± 1.04	2.69 ± 0.85	2.69 ± 0.26	2.43 ± 0.26
R2^*^ [s^−1^]	18.87 ± 0.52	21.61 ± 0.66	9.17 ± 1.66	5.30 ± 1.01	2.73 ± 0.18	3.05 ± 0.15

The intra-subject CoV in maps with acquired B1+ map correction was compared with maps derived from a data-driven B1+ correction (see Figure 3B). Using the acquired B1+ field correction resulted in lower intra-subject CoV of the R1 and PD contrasts in the gray (R1: −33.3%, *p* = 0.04; PD: −49.7%, *p* = 0.007) and white matter (R1: −46.0%, *p* = 0.03; PD: −62.0%, *p* = 0.005). Additionally correcting for Gibb's ringing resulted in a lower intra-subject CoV (*p* < 0.001) of all contrasts in gray/white matter (PD: −12.2/-14.0%, MT: −11.6/-9.2%, R1: −8.0/-5.3%, R2^*^: −13.3/-12.1%).

Figure 3A (lower row) shows the intra-subject variability of individual atlas regions in an example slice for a typical volunteer and Table 2 presents the group-averaged intra-subject CoV of 15 ROIs with high clinical relevance from the Neuromorphometrics atlas alongside volume (data for all regions can be found at https://clinicalmpm.github.io). Intra-subject CoV within atlas regions follows the same pattern as in the tissue masks: PD has the lowest CoV (2.09 ± 1.07%), followed by R1 (4.00 ± 1.20%), MT (6.26 ± 2.54%), and R2^*^ (9.45 ± 3.43%). White and deep gray matter regions are more stable compared to the cortical regions. Using the data-driven B1+ inhomogeneity correction without correcting for Gibb's ringing resulted in poorer stability in all atlas regions (https://clinicalmpm.github.io).

**Table 2:** Mean and standard deviation of intra-subject CoV (scan-rescan) and volume of 15 example atlas regions with high clinical relevance (see remaining atlas regions as sortable table at https://clinicalmpm.github.io).

**ROI name**	**PD CoV [%]**	**MT CoV [%]**	**R1 CoV [%]**	R2^*^ CoV [%]	**ROI volume [cm^**3**^]**
**White matter**					
Cerebral WM	1.24 ± 0.21	3.54 ± 0.91	2.80 ± 0.87	5.31 ± 0.95	556.2
Cerebellum WM	1.81 ± 0.41	4.14 ± 0.82	3.13 ± 1.22	8.23 ± 2.03	33.1
**Deep gray matter**					
Thalamus	1.34 ± 0.22	3.85 ± 1.22	2.71 ± 0.49	6.51 ± 1.11	20.7
Putamen	1.21 ± 0.21	3.02 ± 0.80	2.40 ± 0.48	5.29 ± 1.31	11.5
Hippocampus	1.76 ± 0.38	5.00 ± 1.50	3.42 ± 0.86	10.51 ± 2.25	9.5
Caudate	1.22 ± 0.18	3.59 ± 0.78	2.74 ± 0.85	5.95 ± 0.99	8.6
Pallidum	1.38 ± 0.36	3.39 ± 1.02	2.58 ± 0.55	4.26 ± 0.96	3.8
Amygdala	2.16 ± 0.46	5.14 ± 1.46	3.47 ± 0.84	14.56 ± 3.65	2.5
**Cortical areas**					
Precentral gyrus	1.52 ± 0.44	6.43 ± 2.43	3.82 ± 1.45	7.15 ± 1.59	40.5
Postcentral gyrus	1.73 ± 0.54	7.68 ± 3.53	4.55 ± 1.93	8.38 ± 2.25	34.4
Angular gyrus	1.53 ± 0.48	5.88 ± 2.70	3.99 ± 1.40	7.35 ± 2.11	29.9
Supplementary motor cortex	1.40 ± 0.26	5.78 ± 1.45	3.66 ± 1.34	6.40 ± 1.13	16.0
Occipital fusiform gyrus	2.01 ± 0.55	5.74 ± 2.46	3.67 ± 0.86	10.38 ± 3.37	10.6
Occipital pole	2.92 ± 1.22	8.81 ± 6.49	5.25 ± 2.45	9.43 ± 3.27	9.3
Parahippocampal gyrus	2.72 ± 0.81	6.24 ± 2.67	4.21 ± 1.49	12.97 ± 2.90	7.6

Inter-subject CoV followed a similar pattern as the intra-subject CoV across atlas regions (https://clinicalmpm.github.io): PD has the lowest CoV (2.02 ± 1.82%), followed by R1 (5.12 ± 2.17%), MT (9.33 ± 4.18%) and R2^*^ (9.04 ± 5.61%). Across atlas regions, no differences between intra- and inter-subject variability were found for PD (*p* = 0.64) and R2^*^ (*p* = 0.55) while the inter-subject CoV was slightly higher for MT (by a factor of 1.61; *p* < 0.001) and R1 (by a factor of 1.35; *p* < 0.001).

## Discussion

We present an acquisition and post-processing protocol for quantitative MPM of the brain optimized for longitudinal clinical studies. The acquisition protocol is fully based on manufacturer sequences certified for clinical use and can be immediately translated into clinical protocols without concerns about patient safety or insurance issues. A standard head coil (20 channels) was used for acquisition as it is more readily available in the clinic (e.g., compared to 64-channel coils). Further, the optimized protocol is fully compatible with a wide range of 3-Tesla scanners with less performant gradient systems.

### Multi-Resolution Analysis

The analysis of MPM data acquired with isotropic voxel sizes between 1 and 2.7 mm showed the expected increase in stability with increasing voxel size. The stability of the 1.6 mm protocol was very close to the optimum where physiological noise dominates. A further increase in voxel size would not be expected to increase sensitivity and would rather have a negative impact on the anatomical details that can be resolved. Given the envisioned clinical application, all maps were visually inspected by a neuroradiologist (M.S.) and MPM maps with voxel resolutions higher than 2 mm were confirmed to be of good quality and suitable for most clinical and diagnostic purposes. One example is the detection of multiple sclerosis white matter lesions, which are larger than 3 mm in the long axis (Filippi et al., 2019) and could be well-resolved at these resolutions. The fast MPM protocol with 1.6 mm resolution was confirmed to be suitable for our multi-subject analyses.

### Multi-Subject Analysis

Intra-/inter-subject variability for all four quantitative maps were found to be good both in gray and white matter as well as across all regions in the Neuromorphometrics atlas. Of note, intra-/inter-subject variability of all atlas regions were very comparable, suggesting that the optimized protocol is useful for both cross-sectional and longitudinal clinical studies with similar sensitivity. The intra-subject CoV of all contrasts at 1.6 mm isotropic resolution are roughly 3 times smaller than the values reported by Weiskopf et al. for their 20-min protocol with 1 mm isotropic resolution (Leutritz et al., 2020), even without Gibb's ringing correction. Therefore, in order to achieve the intra-subject CoV of our optimized 1.6 mm protocol, patients would have to be scanned for an estimated 3^2^ = 9 times longer (~3 h) using the 1 mm isotropic protocol by Weiskopf et al. which is not feasible for clinical studies. The mean and intra-/inter-subject CoV of all maps in all 60 atlas regions and the gray and white matter masks are available online (https://clinicalmpm.github.io) for use by clinical researchers for sample size and effect size estimations (Sullivan and Feinn, 2012) for microstructural changes in specific ROIs using MPM.

An apparent spatial distribution of longitudinal variability in all four contrasts was identified, where white and deep gray matter regions had lower CoV compared to cortical gray matter. This was particularly apparent in the gray/white matter contrast, where white matter CoV was 50% lower than gray matter. Given that cortical gray matter has a higher amount of vasculature compared to white and deep gray matter (Bernier et al., 2018), which could lead to artifacts from blood and cerebrospinal fluid (Havsteen et al., 2017), we hypothesize that this could contribute to the observed higher variability of MPM parameters in cortical regions as this has also been shown in functional MRI (Mueller et al., 2013; Chamberland et al., 2017; Bernier et al., 2018; Provencher et al., 2018). The effect of vascularization on MPM has, to our knowledge, not yet been investigated and represents a potentially interesting area for future optimization.

We recommend correcting for Gibb's ringing artifacts. Removing the typical oscillatory artifacts surrounding sharp tissue boundaries not only increases the overall image quality for diagnostic purposes but also increases temporal stability as the destructive effect of Gibb's ringing is amplified due to subject motion. Gibb's ringing had very little influence on the absolute values of MPM maps, while stability significantly increased. While correcting for Gibb's ringing increased stability in all maps, the effect was less strong for R1. A possible interpretation of this would be that the other three maps (PD, MT, and R2^*^) are derived from raw data with high (unsuppressed) CSF signal and therefore particularly benefit from Gibb's ringing correction. We suggest that high-resolution MPM studies trying to resolve cortical layers (Trampel et al., 2019) might especially benefit from Gibb's ringing correction.

Given the need for short acquisition times in the clinical setting, we tested which contrasts benefit from the additional scan time (~2 min) required to acquire a B1+ map. Using the acquired B1+ maps increased the stability for PD and R1 with slight deviations in absolute R1 and MT values as expected, with higher deviations in R1 than MT (Callaghan et al., 2014; Leutritz et al., 2020). As expected, R2^*^ quantification is not affected by B1+ mapping (Tabelow et al., 2019). Hence, for clinical studies with particularly limited scan time or in populations where patient compliance is challenging, it may be possible to reduce the total scan time to 7 min, sacrificing some stability in R1 and PD. Alternatively, the MPM protocol may be extended to correct B1- inhomogeneities using the short (< 1 min) scan presented by Leutritz et al. (2020), according to the work of Papp et al. (2016).

In line with previous reports (Weiskopf et al., 2013; Leutritz et al., 2020), R2^*^ had the lowest stability in the current study. Possible measures to improve the stability may be to correct for B0 inhomogeneities by adapting sequences as described by Baudrexel et al. (2009) or by using the existing phase images acquired with the MPM protocol (Cohen-Adad et al., 2012). Phase images may also be used to derive quantitative susceptibility maps (Acosta-Cabronero et al., 2018). Another potential extension would be to calculate the macromolecular proton fraction from the acquired MPM maps (Yarnykh, 2016), to acquire an additional marker of myelination with high stability (Yarnykh et al., 2020).

### Important Considerations for Clinical Applications

The real benefit of quantitative measures is the ability to make statistically valid comparisons and interpret effect sizes across brain areas, subjects, species and sites (Sullivan and Feinn, 2012; Weiskopf et al., 2015; Chen et al., 2017). Therefore, excellent reporting standards are required, including a-priori power calculation and effect sizes. This is a well-described problem in the field of functional MRI research (Sullivan and Feinn, 2012; Chen et al., 2017; Nichols et al., 2017). We only found two MPM papers that reported absolute values and effect sizes (Grabher et al., 2015; Lommers et al., 2019). Weiskopf et al. (and the current study) provide important reference values for mean and standard deviation and intra-/inter-subject reliability. This should enable clinical researchers to calculate detailed power analyses and report effect sizes that can be interpreted in the context of future studies as well as enable the implementation of their findings in the clinic.

### Limitations

Although the intra-subject variability reported in the current study is good (0.2–10%), it is higher than recently reported intra-subject variability in T1, T2^*^, and PD (Gracien et al., 2020). This might be explained by several factors: First, a different B1+ inhomogeneity correction method was used, which can have a large effect on stability of qMRI parameters as demonstrated in the current study. Second, the explicit use of non-manufacturer sequences allowed for higher optimization than was possible in the current study and may explain some differences. Further, as recently highlighted (Leutritz et al., 2020), PD maps are calibrated to 69% in the white matter (Tabelow et al., 2019), which may have led to an underestimation of variability. The current study aimed to implement sequences for MPM that were fully certified for clinical use and based on manufacturer sequences, allowing for safe and immediate translation into clinical protocols (not just research centers). Hence, we were unable to implement many experimental sequence adjustments (Callaghan et al., 2019) that could further optimize the stability of the qMRI parameters. Finally, this was a single-center study and a multi-center validation of the current protocol, similar to Weiskopf et al. (2013) is warranted for future research.

### Conclusions

In conclusion, we present an optimized 7–9 min MPM protocol at 1.6 mm isotropic resolution that is fully certified for clinical use, is suitable for diagnostic purposes and can be easily translated into clinical protocols. All scan protocols, processing scripts and variability data are available for download at https://clinicalmpm.github.io. We show that a lower resolution and correcting for Gibb's ringing in the preprocessing stage robustly produces high-quality PD, MT, R1, and R2^*^ maps with absolute values comparable to those previously reported in healthy participants using MPM (Weiskopf et al., 2013; Callaghan et al., 2014; Lommers et al., 2019). The optimized MPM protocol has excellent intra-subject variability both in tissue classes and individual atlas regions. The stability of our optimized protocol is 2–3 times higher than for widely used 1-mm MPM protocols (Weiskopf et al., 2013; Callaghan et al., 2014; Grabher et al., 2015; Ziegler et al., 2018; Lommers et al., 2019; Leutritz et al., 2020; Taubert et al., 2020) and was achieved in less than half the acquisition time. Given the high effect sizes reported in former MPM studies (Grabher et al., 2015; Lommers et al., 2019), the optimized protocol is expected to have high sensitivity to detect subtle changes related to disease or treatment effects even at the single-subject level, making the current protocol a candidate for rare diseases and personalized medicine approaches.

## Data Availability Statement

The datasets presented in this study can be found in online repositories. The names of the repository/repositories and accession number(s) can be found in the article/supplementary material.

## Ethics Statement

The studies involving human participants were reviewed and approved by Charité Ethics Committee, Charité University Medicine Berlin, Berlin, Germany. The patients/participants provided their written informed consent to participate in this study.

## Author Contributions

GC and SHe contributed to the conception and design of the study, performed statistical analysis and MRI processing, and wrote the first draft of the manuscript. All authors contributed substantially to interpretation of the data and revised the manuscript critically for intellectual content and have approved the submitted version.

## Conflict of Interest

The authors declare that the research was conducted in the absence of any commercial or financial relationships that could be construed as a potential conflict of interest.
